# Heart Rate Variability During Short-Term Electroencephalography

**DOI:** 10.7759/cureus.114073

**Published:** 2026-08-06

**Authors:** Miles S Evans, Catarina De Marchi Assunção, Vishwanath Sagi

**Affiliations:** 1 Department of Neurology, University of Louisville School of Medicine, Louisville, USA

**Keywords:** autonomic nervous system (ans), electrocardiography (ecg), electroencephalography (eeg), frequency domain analysis, heart rate variability, neurology and epilepsy, parasympathetic nervous system, prognostic tools, sleep stages, time domain analysis

## Abstract

Introduction: Heart rate variability (HRV) is a measure of the function of the brainstem nuclei that modulate heart rhythm through the parasympathetic and sympathetic nervous systems. HRV is reduced in many health conditions. It is a predictor of increased mortality in patients who have had myocardial infarction, and of sudden death in persons with epilepsy. Methods: We measured HRV in the single-channel electrocardiogram (ECG) of 103 patients undergoing a short electroencephalogram (EEG) of 20 to 90 minutes' duration, whose EEG results were normal.

Results: We found that despite the normal EEG results of the patients, their measures of HRV were reduced compared to normal values. The single most widely used measure of HRV is the square root of mean squared differences of successive heartbeats (RMSSD), which was 28.1 ± 21.8 in our subjects. The high frequency (HF) band was specifically affected. In these patients, HRV was only slightly affected by state of wakefulness, and was stable during wakefulness, N1 sleep, and N2 sleep. A significant reduction in HRV with age was also observed in our subjects. There was a non-significant trend toward lower HRV in our female subjects.

Conclusion: In patients selected by their clinicians to undergo EEG, HRV is reduced. Our results indicate that HRV during the first five minutes of EEG, regardless of sleep state, is a reliable measure of HRV in patients undergoing short-term EEG. We conclude that a normal EEG result does not predict normal HRV and that routine measurement of HRV during EEG could provide useful prognostic information for patients undergoing EEG.

## Introduction

Heart rate (HR) is not entirely steady. In normal persons, HR increases during exercise and decreases at rest, but HR variability occurs on a moment-to-moment basis even at rest. Resting HR varies at short time intervals due to respiration (respiratory sinus arrhythmia) and blood pressure mediated through the parasympathetic nervous system. Resting HR is modulated on longer time scales due to circadian rhythms and sympathetic nervous system activity [1]. Cardiovascular parasympathetic activity is integrated by brain medullary cardiovascular nuclei, including the nucleus tractus solitarius, nucleus ambiguus, and dorsal motor nucleus of the vagus, and conducted through vagus nerve fibers to the heart sinoatrial (SA) node [2,3]. The pontine Kölliker-Fuse nucleus, primarily concerned with respiration, strongly influences respiratory sinus arrhythmia through projections to the medullary cardiovascular nuclei [4]. Reduced heart rate variability (HRV) is associated with many diseases and conditions. The most well-known association is with prognosis after myocardial infarction (MI): survivors of MI with higher HRV have a better prognosis than those with low HRV [5].

Recent research has shown that epilepsy is associated with increased rates of cardiac death [6] and reduced HRV is associated with sudden unexpected death in epilepsy (SUDEP) [7]. Routine inter-ictal electroencephalogram (EEG) is frequently done in patients with epilepsy, and single-channel electrocardiogram (ECG) is usually performed simultaneously with the EEG. The ECG is occasionally useful for detecting tachy- or bradyarrhythmias but is used mainly to detect ECG artifact in the EEG channels. Potentially, the ECG channel in EEGs could also be useful for analysis of HRV, which may have diagnostic or prognostic value in persons undergoing EEG.

HRV is a continuously changing function of brain and autonomic nervous system action on the heart. Since HRV is a vagally mediated reflex controlled by brainstem nuclei, it might be regarded as a kind of brain rhythm, so changes in HRV might be expected when other disturbances of brain rhythms are present on EEG. Long-term HRV has been studied using Holter monitoring [8] and HRV in patients with epilepsy has been studied using Holter monitoring [9] and polysomnography [10], and in other specific neurological conditions such as SUDEP [7], but there are no previous studies of HRV in a general population of patients undergoing routine short-term EEG, even though single channel ECG is routinely recorded during these studies.

Accurate measurement and interpretation of HRV is non-trivial, but there are well-established guidelines for it [8]. EEG testing is usually done with the patient supine and at rest for prolonged periods, from minutes to days. This is a technically favorable situation for recording resting ECG, so HRV measurements during it are potentially highly informative. The purposes of this study are to determine whether HRV can be accurately measured from the single ECG channel during ordinary short EEGs, to estimate the HRV values found in the population usually undergoing an EEG, and to determine whether having a normal EEG is likely to imply also having normal HRV.

## Materials and methods

We did a retrospective single-center study of single-channel ECG in 103 normal adult EEGs, which at our institution is about two weeks of normal routine short-term EEGs. EEGs were 20 to 90 minutes in duration. They were recorded in the University of Louisville Health System, either in a hospital (University of Louisville Hospital or Jewish Hospital) or in the University of Louisville Neurology Clinic. A total of 23 were normal inpatient studies, and 80 were normal outpatient studies. EEGs were all clinical studies done on adult patients at the order of their treating physicians, not for research. Before this study, EEG records were stripped of all personal identifying information and clinical information. Information remaining after de-identification was age, sex, the interpretation of the clinical reader that the study was normal, status as inpatient or outpatient, and the EEG data itself. The full EEG report, which might contain personal identifying information, was not available. We included only adult patients aged 17 years or older who had normal sinus rhythm. A total of 112 normal EEGs were originally obtained for the study, but nine were rejected because review of the ECG channel revealed that the subject had a cardiac pacemaker (four) or atrial fibrillation (five).

EEGs and the corresponding ECGs were recorded using a Nihon Kohden (Tokyo, Japan) instrument. ECG was recorded with a single lead II, with a right subclavicular electrode and a left lateral chest electrode. Lead II is excellent for recording heart rhythm, but poor for recording ischemic ST segment changes [11]. Digitization rate was 250 Hz. For EEG review, a low-pass filter was set at 70 Hz, a high-pass filter at 1 Hz, and when desired by the reviewer, a notch filter at 60 Hz.

**Figure 1:**
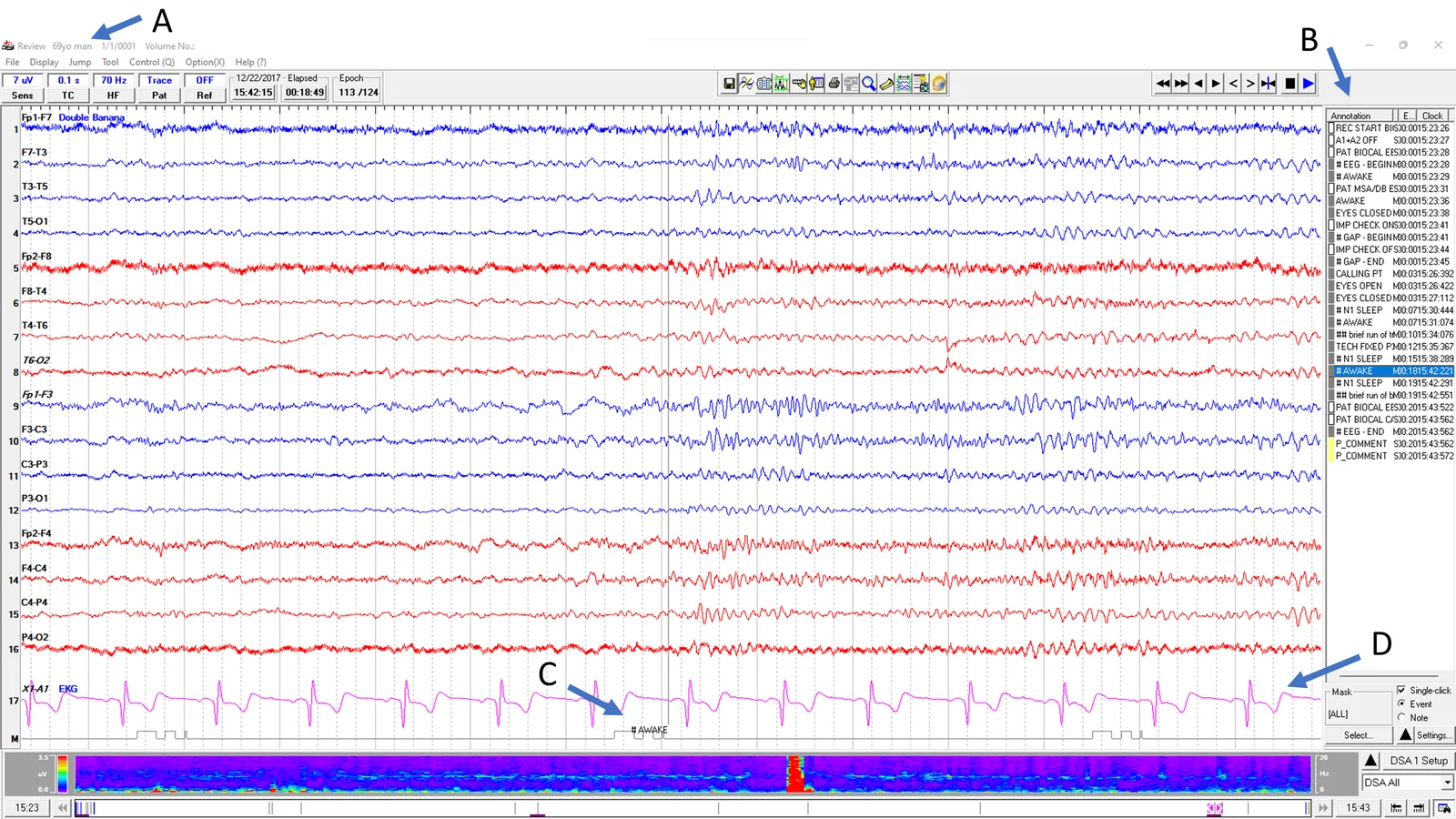
Marking state changes in an EEG. Relevant events and state changes were manually marked on de-identified EEGs by the investigators, and the marks, times, and ECG trace were exported for further analysis. (A) EEGs were de-identified. (B) Events of interest in the EEG recording were marked with a standardized comment beginning with “#”. (C) In this EEG page, the transition from sleep to awake was marked with the comment “# AWAKE”. (D) The single-channel ECG was exported to a text file in American Standard Code for Information Interchange (ASCII) format.

We were interested in whether HRV changes with EEG states of wakefulness and sleep. Interpretation of EEG ordinarily includes a statement of the features of the waking EEG, for example the presence or absence of a posterior dominant alpha activity when eyes are closed, and a note of whether sleep occurred during the study and if so, the stages achieved, perhaps with a statement of the features of that stage, for example the presence of sleep spindles in stage N2. However, the clinical reader does not annotate the EEG to note the times of onset and offset of waking, sleep, nor their durations. For us to correlate state of wakefulness or sleep with HRV, one of the authors of the study (MSE) reviewed each EEG and marked the time of onset and offset of these EEG features: awake, stage N1 sleep, stage N2 sleep, stage N3 sleep, rapid eye movement (REM) sleep, hyperventilation, and photic stimulation. The times these states occurred were marked using a standardized set of comments (# AWAKE, # N1 SLEEP, # N2 SLEEP, # N3 SLEEP, # REM SLEEP, # HV BEGIN, # HV END, # PS BEGIN, # PS END, # EEG BEGIN, # EEG END) (Figure 1) that could later be analyzed by a computer program. Stages N3 sleep and REM sleep were marked but occurred too rarely in short EEGs to analyze. The activation procedures of hyperventilation and photic stimulation were done on too few patients to analyze. The patients were usually in the awake state during activation procedures, but the EEG data during the activation procedure were not included in the analysis of HRV for awake or sleep states.

It is standard for HRV to be calculated using time intervals of five minutes for “short-term” HRV and 24 hours for “long-term” HRV [8]. We used five minutes for this study, so HRV for a specific state was reported only if an awake or asleep state lasted a full five minutes. If the state lasted 10 minutes, two successive HRV values could be measured. If a stage lasted less than five minutes, the HRV data were not included in the analysis for that state. This requirement further reduced the number of studies available for HRV analysis by state: for example, of the 103 subjects who had a normal sinus rhythm, only 95 had a full five minutes of awake state EEG.

**Figure 2:**
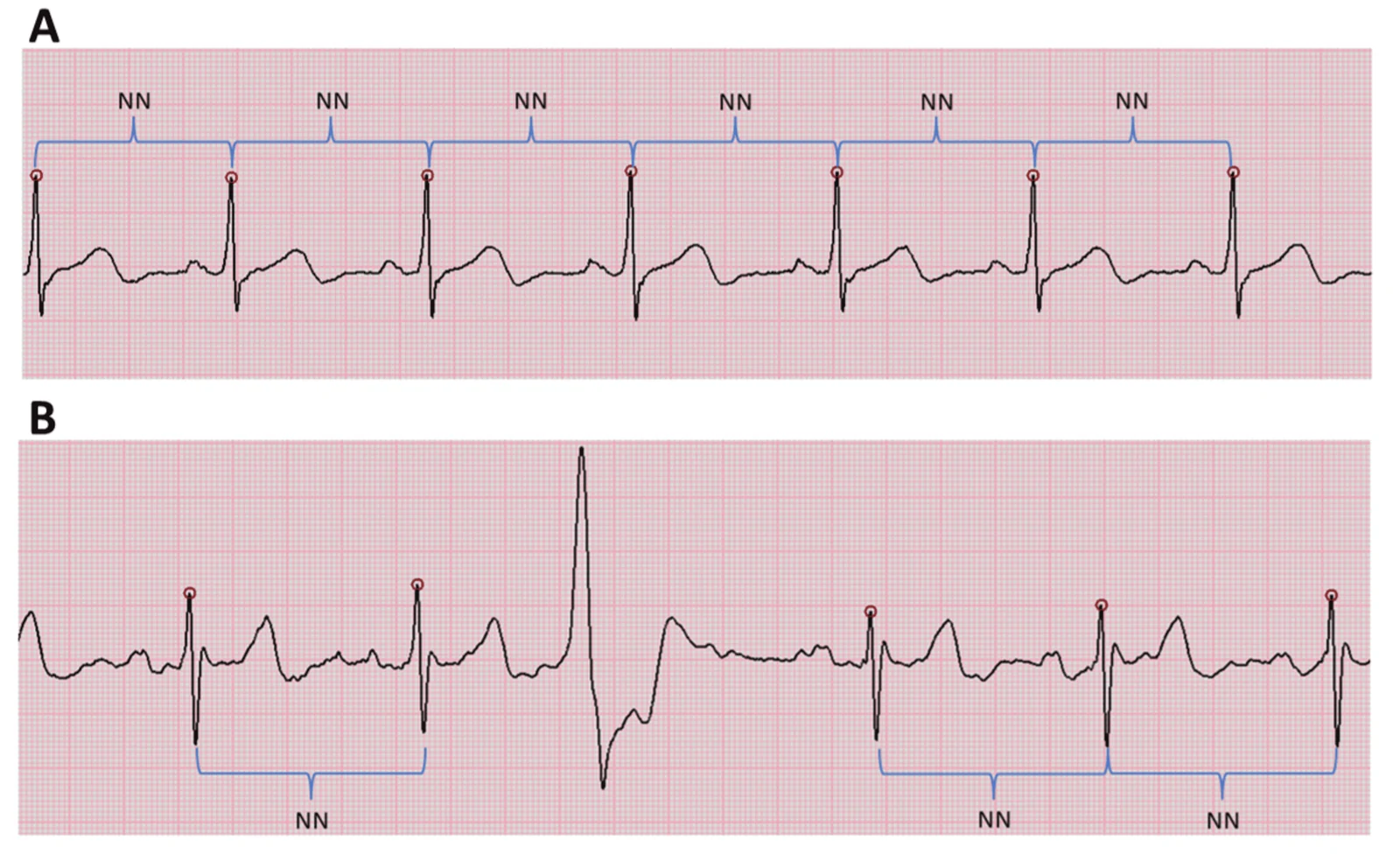
Measurement of NN intervals. (A) A modified Pan Tompkins algorithm was used to place fiducials (maroon circles) at the peak of the R waves. All the beats are normal, so the measured RR intervals are NN (normal-to-normal) intervals. (B) This person had a premature ventricular contraction (PVC, the third QRS complex), and the RR intervals surrounding it are therefore not normal. A fiducial that our computer algorithm placed at the PVC peak was manually deleted by the investigator, and only the remaining NN intervals were measured. The scale in the top panel is 500 µV x 200 ms, and in the bottom panel is 200 µV x 200 ms.

We used the American Academy of Sleep Medicine (AASM) criteria for sleep staging [12]. AASM sleep stages are determined in 30-second epochs. After the reviewer marked sleep stages, data from the single-lead ECG channel, filtered from 1 to 70 Hz, were output to an American Standard Code for Information Interchange (ASCII) text file. The ASCII file was read by a computer program written in Python 3.9 (Python Software Foundation, Wilmington, DE). The program allowed the reviewer to visualize the ECG trace, page back and forth through it, visually examine each QRS complex, and mark the desired fiducial point, which was the maximum or minimum of the R wave (Figure 2). The reviewer could, if desired, manually mark each fiducial point, but this would be excessively laborious, since a 60-minute ECG of a patient with a heart rate of 70 beats per minute would contain 4200 fiducial points. Instead, we opted for a mixture of automatic and manual marking of R waves, so the primary task of the reviewer was to quickly review and verify or correct the marked records, rather than to find the peaks and make the marks.

There are several accepted methods of automatically detecting R waves in ECG data. We used a Python package containing eight ECG heartbeat detection algorithms [13]. The package implements these algorithms: Hamilton, Pan and Tompkins, Christov, Engelse and Zeelenberg, stationary wavelet transform, and Pan and Tompkins with matched filter. We found the Pan and Tompkins with matched filter most useful, so we used it most often, but certain ECGs did not work well with that algorithm, and one of the other seven was used in those cases.

Although all the detectors worked well at computing beat-to-beat intervals, none of them were good at identifying the actual peak or trough of the R waves. The algorithm might very accurately and consistently mark a point in the QRS complex other than the peak of the R wave, and therefore give a result with correct intervals, but marking a point other than the peak made it very slow for a person to visually review it for accuracy. To allow quick and accurate human review, we combined the detector algorithm with a peak/trough detector algorithm, adjustable high- and low-cut filters, and an optional peak-enhancement algorithm so as to accurately mark the positive peak or negative trough of the R wave as the correct fiducial point (Figure 2A). In all ECGs, every fiducial point was reviewed by a human reviewer and determined to be correct.

All detectors were prone to incorrectly marking premature beats and artifacts, which were both relatively common in our EEGs. To avoid these errors, each marked fiducial point was corrected manually whenever necessary (Figure 2B). The final output of the ECG review program was a text file of investigator-confirmed RR intervals that included only normal-to-normal (NN, not premature) beats with the time of the occurrence of that NN interval from the beginning of the EEG.

The file of NN intervals and times of occurrence was then combined with comments from the EEG into a single file having the times of onset and offset of wakefulness, sleep stages, hyperventilation, and photic stimulation. These combined data were then used to determine HRV during the different states found in the EEG.

To determine the standard HRV parameters, we used a Python package supplied by Aura Healthcare [14]. This package allows analysis of time-domain, frequency-domain, and non-linear domain HRV features. Statistics were done using the SciPy (scipy.stats) package for Python. We used analysis of variance (ANOVA), two-factor analysis of covariance (ANCOVA), Pearson's correlation coefficient, and Student's t-test to assess various group differences in the time-domain measures, including root mean square of successive differences (RMSSD), standard deviation of normal-to-normal intervals (SDNN), and normal-to-normal interval (NNI), and in the frequency-domain measures, including high frequency (HF), low frequency (LF), and LF/HF ratio. Except for RMSSD, skewness and kurtosis data for time-domain measures showed near-perfect normality in most cases (-0.5 to +0.5) or moderate departure (-1.0 to -0.5 or +0.5 to +1.0) in a few cases. Skewness and kurtosis for individual statistical analyses confirmed normality in specific tests. RMSSD, HF, LF, and LF/HF ratio all have a strong positive skew, so these measures were log-transformed for statistical analyses, which resulted in near-perfect normality by skewness and near-perfect normality or moderate departure by kurtosis. In one analysis where variances of groups were clearly unequal (inpatient versus outpatient RMSSD), we used an unequal variance test version of Student's t-test.

We compared our age-dependent RMSSD results to those of two previous studies of healthy normal subjects by Agelink et al. [15] and Abhishekh et al. [16]. Agelink et al. [15] reported mean RMSSD data by decade in log values, which we transformed to the usual non-log units. Abhishekh et al. [16] reported their data only in a figure. We digitized the points in their figure using Engauge Digitizer (https://markummitchell.github.io/engauge-digitizer/) and tabulated them using the same age ranges as Agelink et al. [15].

This study was approved by the University of Louisville Human Subjects Committee.

## Results

Five minutes of ECG in a supine, relaxed state is the most common type of ECG used for HRV analysis and is the testing condition for which normal values are most available. To maximize comparability of our results to published normal values, our primary analysis was simply the first five minutes of the EEG. HRV analyses are standardized and are classified into three basic types: time domain measures, frequency domain measures, and non-linear measures.

Time domain HRV

**Figure 3:**
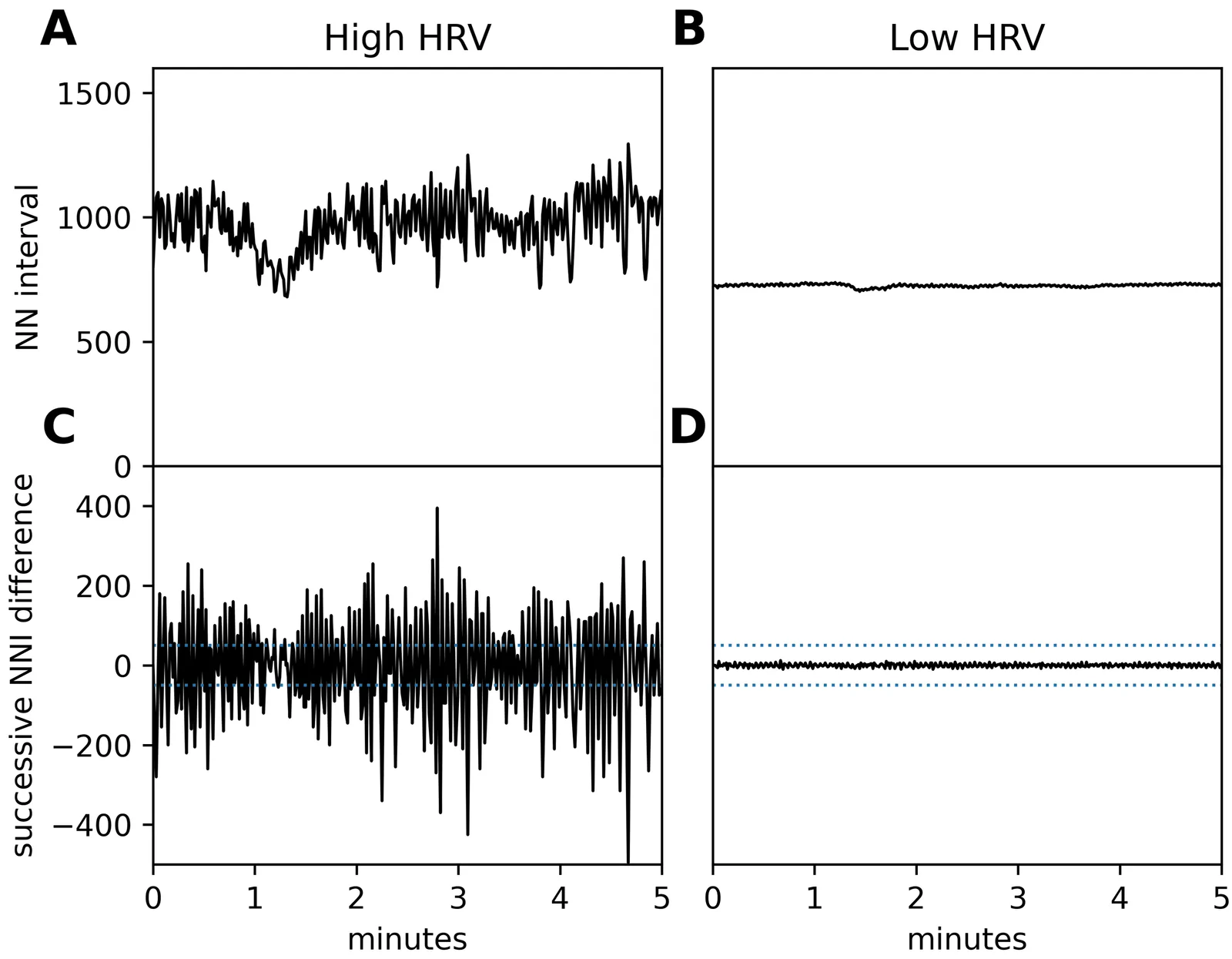
Examples of heart rate variability (HRV) in five-minute tachograms of awake EEG. Standard HRV measurements are calculated over five-minute epochs. These HRV examples are from the first five-minute awake epoch. (A) This example is from a 21-year-old woman who had the highest HRV of all our subjects. Her minimum heart rate (HR) during these five minutes was 46 beats per minute, maximum was 88, and RMSSD was 133 ms. (B) This example is from a 61-year-old woman who had the lowest HRV of all our subjects. During this five-minute epoch, her minimum HR was 81, maximum was 85, and RMSSD was 4.3 ms. (C) A plot of the successive NN interval differences for the subject in (A) and (D) the subject in (B) emphasizes the marked differences in HRV in these two subjects. The 21-year-old woman had 218 occurrences of a greater-than-50 ms difference between successive NN intervals (NNI 50 = 218), while the 61-year-old woman had 0 occurrences (NNI 50 = 0). RMSSD: root mean square of successive differences; NN: normal-to-normal; NNI: normal-to-normal interval.

Figure 3 illustrates the range of HRV found in our subjects. It shows the tachogram (NN intervals) during the first five minutes of ECG (EEG awake state) in two subjects with normal EEGs. One subject (Figures 3A, 3C) had the highest HRV of all our subjects, and the other had the lowest (Figures 3B, 3D). The five-minute tachograms for each study subject in each state were analyzed for HRV.

Time domain measures of HRV describe the statistical variability of the NN intervals during a time epoch. Table 1 shows a list of these measures, along with normal values for short-term recordings of five minutes duration. The most useful and accepted time domain measure is the RMSSD between successive NN intervals in a five-minute epoch. There are other time domain measures, such as the number of successive NN intervals with a greater than 50 ms difference, as shown in Figure 3, but time domain measures are all correlated with each other.

**Table 1:** Time domain measures of HRV. Heart rate variability (HRV) was measured during the first five minutes of EEG during the awake state for 103 subjects. HRV as measured by SDNN and RMSSD was low in patients getting EEGs compared to normal values. Normal values are from a comprehensive review of normal values for short-term ECG by Nunan et al. [17]. Because the number of subjects was large, SDSD and RMSSD are the same when written to one decimal place.

Variable	Description	Subjects, Mean ± SD	Normal values, Mean ± SD	Range
Mean NNI (ms)	Mean normal-to-normal interval	846.2 ± 141.4	926 ± 90	785-1160
SDNN (ms)	Standard deviation of the normal-to-normal intervals	39.47 ±25.2	50 ± 16	32-93
SDSD (ms)	Standard deviation of the differences between normal-to-normal intervals	28.1 ± 21.8		
NNI50 count	The number of successive normal-to-normal intervals that differ by more than 50 ms	26.75 ± 47.4		
pNN50 (%)	The percentage of successive normal-to-normal intervals that differ by more than 50 ms	8.5 ± 14.9		
RMSSD	Square root of mean squared differences of successive normal-to-normal intervals	28.1 ± 21.8	42 ± 15	19-75

We compared the HRV results in our subjects to the normal values reported in a large review by Nunan et al. [17] of published studies involving 21,438 participants. Although our study subjects had normal EEGs, they had low HRV as assessed by time domain measures (Table 1). Mean NNI (i.e., heart rate) was comparable to the mean of normal subjects, but our subjects had lower RMSSD and SDNN. RMSSD is the most accepted single measure of time-domain HRV, and the mean value of that and of SDNN in our subjects (21.6) was approximately -1 SD lower than the normal mean reported by Nunan et al. (i.e., 42) [17].

Influence of EEG state on time domain measures of HRV

**Figure 4:**
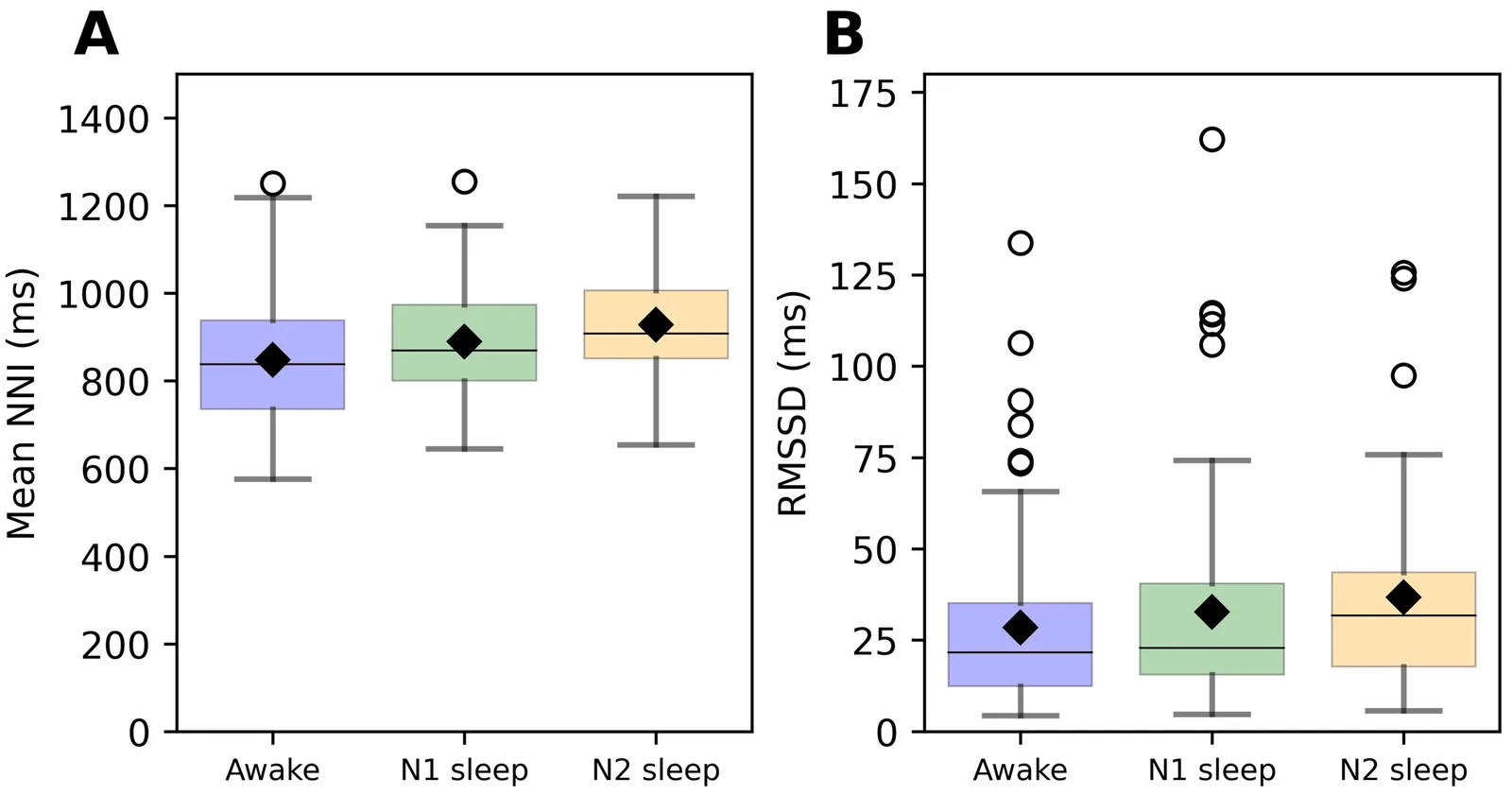
Effect of wakefulness and sleep on HRV. (A) There was a statistically significant increase (p < 0.01) in NN intervals (decrease in heart rate) during sleep (N = 95 awake, 76 N1 sleep, 42 N2 sleep). (B) There was a statistically nonsignificant trend for an increase in HRV (RMSSD) during sleep. Box and whisker plots in this and other figures show the mean (black diamond), median (horizontal line), 25th percentile (box bottom), 75th percentile (box top), 25th percentile – 1.5x interquartile range (lower whisker), 75th percentile + 1.5 x interquartile range (top whisker), and outlier data points (circles). HRV: heart rate variability; RMSSD: root mean square of successive differences; NN: normal-to-normal.

There are published studies of the influence of sleep on HRV, but the studies have been done with 24-hour ECGs [18]. Sleep during a short EEG is necessarily brief. Stage N2 sleep is usually the deepest attained. In our EEGs, some patients did achieve stage N3 or stage REM sleep, but they were too brief or too few subjects attained those stages to analyze. However, enough subjects had five minutes of awake, stage N1 sleep, and stage N2 sleep to analyze (Figure 4).

In our subjects, as expected, there was a trend toward an increase in NN interval (slowing of HR) in N1 sleep compared to awake, and a further increase in N2 sleep compared to N1 sleep. An increase in NNI during sleep is expected. The change in our subjects was small, but consistent and statistically significant. One-way ANOVA for NNI versus sleep state was significant (F = 5.5, p < 0.01). There was a corresponding but nonsignificant trend toward greater HRV as measured by RMSSD during sleep as compared to wakefulness. RMSSD has a strong positive skew. One-way ANOVA for log-transformed RMSSD versus sleep state was not significant (F = 1.7, p = 0.21).

HRV during sustained time in EEG state

**Figure 5:**
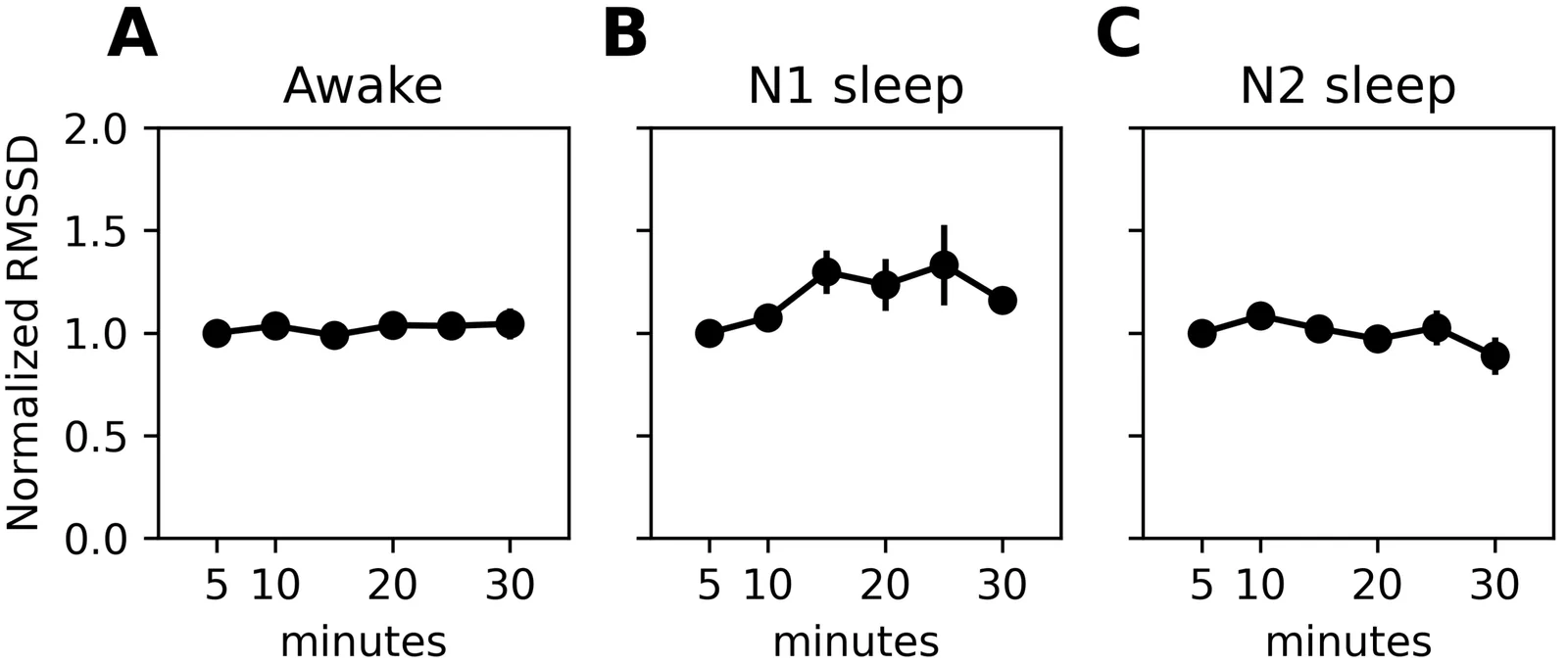
HRV during continuous EEG wakefulness and sleep is relatively stable. (A) Normalized RMSSD is stable during continuously maintained wakefulness. (B) In stage N1 sleep, RMSSD increased slightly, but (C) there was no appreciable change during continuously maintained stage N2 sleep. HRV was analyzed in five-minute segments (0-5 minutes, 5-10 minutes, and so on) and was normalized to the value during the initial 0-5 minutes. Error bars are + and - SEM, and if not shown are smaller than symbol size. HRV: heart rate variability; RMSSD: root mean square of successive differences.

We asked whether HRV is stable in each state during an EEG. To investigate this, we analyzed the subset of subjects who had awake or sleep states of 10 or more minutes (Figure 5). We normalized each five-minute segment to the first five-minute segment in that state for each subject. We found that HRV as measured by normalized RMSSD remained approximately stable in each state. This stability persisted for 25 minutes or more in the awake state, and 20 minutes or more in N1 and N2 sleep. RMSSD during sustained wakefulness was stable, with very small changes that were not statistically significant. One-way ANOVA for the effect of time during the awake state (N = 95) was non-significant (F = 0.36, p = 0.84). The effects of prolonged N1 sleep and prolonged N2 sleep on RMSSD were both relatively small, with a maximum increase of 33 during N1 sleep at 25 minutes, but they were consistent and statistically significant. One-way ANOVA for time effect during N1 sleep (N = 76) was significant (F = 4.23, p < 0.01) and during N2 sleep (N = 76) was also significant (F = 4.3, p < 0.01).

Age and sex correlation to RMSSD and SDSD

**Figure 6:**
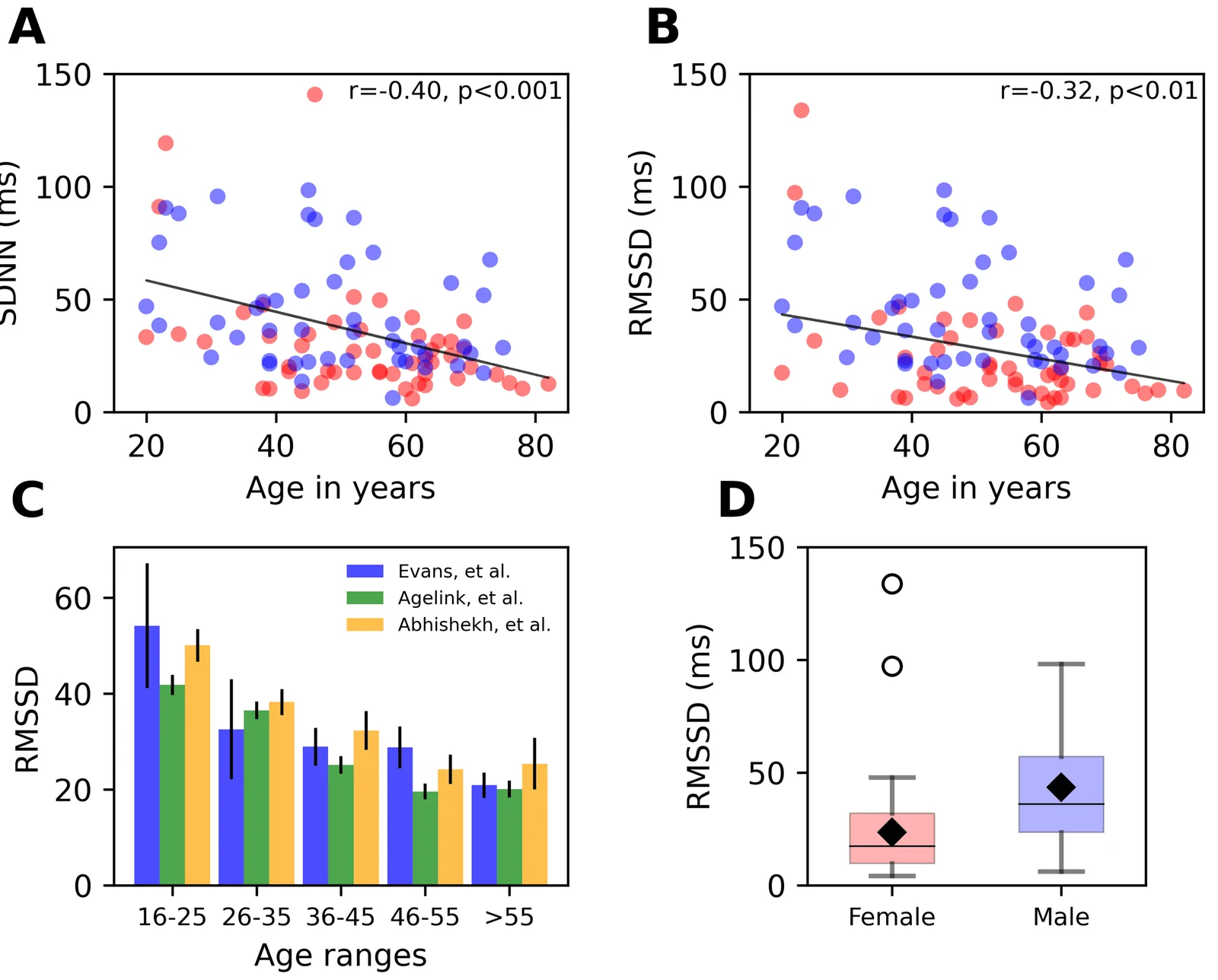
Age and sex relationship for time domain measures of HRV. The values shown in each graph are for the first five minutes of the EEG. There were declines in both (A) SDNN and (B) RMSSD with age. A linear trendline for our subjects is shown with a dotted line. There is a highly significant negative correlation (r) between age and SDNN and between age and RMSSD. In (A) and (B), female subjects are indicated by red and male by blue symbols. (C) The age-dependent results per decade from our subjects having a normal EEG are comparable to those of normal subjects studied by Agelink et al. [15] and Abhishekh et al. [16]. (D) More subjects were female (54) than male (49), and the average age of females (53.4) was greater than that of males (average age = 48.8 years). Female subjects had an overall lower RMSSD than male subjects. Two-factor ANCOVA of age and sex effects on RMSSD showed a highly significant effect of age, but the effect of sex was not statistically significant. HRV: heart rate variability; SDNN: standard deviation of normal-to-normal intervals; RMSSD: root mean square of successive differences; ANCOVA: analysis of covariance.

Previous studies of normal adult HRV showed a relationship between HRV and age, finding reduced HRV with aging. In our study, the time-domain measures of RMSSD and SDNN were both negatively correlated with age (Figures 6A, 6B). The strength of the correlation was moderate, -0.40 for SDNN and -0.32 for RMSSD. RMSSD is positively skewed, so statistics were done on log-transformed RMSSD. The Pearson correlation coefficient for age was highly statistically significant for both SDNN (p < 0.001) and RMSSD (p < 0.01).

There have been two previous relatively large studies of the effects of age on HRV in healthy subjects. Agelink et al. [15] studied HRV in 309 healthy subjects and Abhishekh et al. [16] in 189. Both reported age effects on RMSSD. In Figure 6C, our RMSSD results are compared to the results from their studies. Both the decline with age and the absolute values for RMSSD appear similar for all three studies, suggesting that the overall RMSSD results from our subjects are comparable.

Sex differences in HRV have been described. In our subjects, females tended to have lower RMSSD values than males (Figure 6D). ANCOVA for the effects of age and sex on log-transformed RMSSD showed a highly significant effect of age (F = 10.0, df = 1, p < 0.01) but the effect of sex was not statistically significant (F = 2.26, df = 1, p = 0.19).

Frequency domain measures of HRV

**Figure 7:**
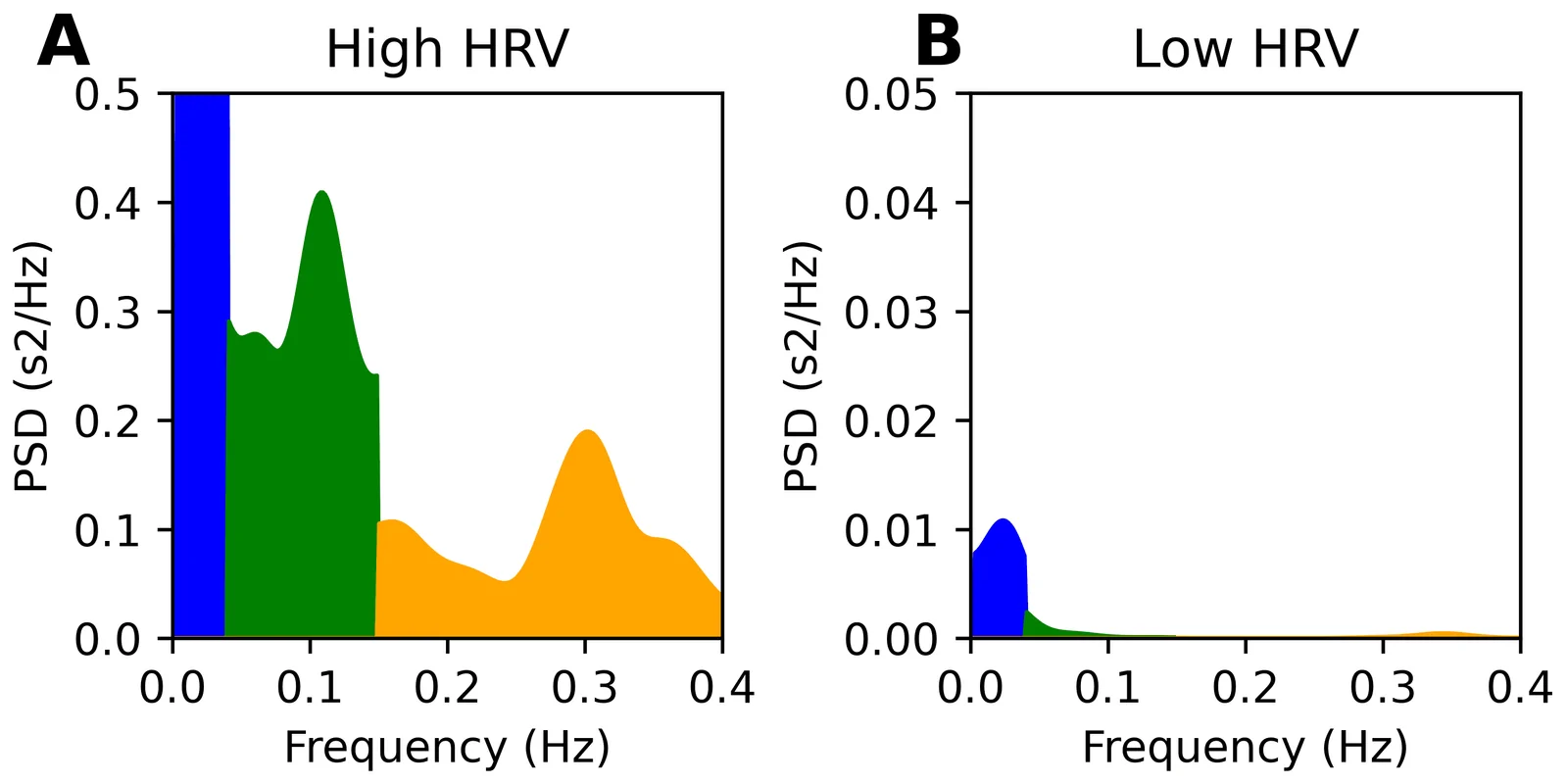
Illustration of frequency domain measures of HRV. These are Welch periodograms for the same two subjects as in Figure 3. (A) For the subject with high heart rate variability (HRV), power in the low frequency (LF, green) band is greater than power in the high frequency (HF, orange) band. The LF/HF ratio is normal. Very low frequency (VLF, blue) power is even higher, but VLF measurement is only considered reliable in 24-hour ECG recordings (VLF power greater than 0.5 is not shown). (B) For the subject with abnormally low HRV, power is very low in all frequency bands. Note the 10-fold difference in scale for the two graphs.

Frequency domain methods classify the rhythmic activity in the tachogram by Fourier analysis of frequency. The different bands are thought to reflect the activity of different physiological processes. The high frequency band, for example, contains the variability due to respiratory sinus arrhythmia when the person is breathing at normal rates. Table 2 shows a list of frequency domain measures, a short description, and the results in our subjects. As a group, in our subjects, power was reduced in a single band, the HF band. The HF band power is 657 ms^2 ^in healthy normal subjects, but only 311 in patients undergoing an EEG. Figure 7 illustrates the HF and LF frequency bands with examples of two subjects, one with normal HRV and one with reduced HRV.

**Table 2:** Frequency domain measures of HRV. HRV was measured during the first five minutes of EEG during the awake state for 95 subjects. Patients getting EEGs, relative to published normal values, have lower power in the HF band. The ratio of low frequency to high frequency power is within the normal range. Normal values are from a comprehensive review of normal values for short-term ECG by Nunan et al. [17]. The ranges are for the mean values in various published studies. HRV: heart rate variability; SD: standard deviation; LF: absolute power in low frequency band; HF: absolute power in high frequency band; LF (nu): relative power in low frequency band, normalized units; HF (nu): relative power in high frequency band, normalized units; LF/HF: ratio of low frequency to high frequency power.

Variable	Description	Subjects, Mean ± SD	Normal values, Mean ± SD	Range
LF (ms^2^)	Power in low frequency band	537 ± 913	519 ± 291	193-1009
LF (nu)	Power in low frequency band	59 ± 21	52 ± 10	30-65
HF (ms^2^)	Power in high frequency band	311 ± 575	657 ± 777	83-3630
HF (nu)	Power in high frequency band	42 ± 21	40 ± 10	16-60
LF/HF (ms^2^)	Ratio of low frequency to high frequency power	2.7 ± 3.6	2.8 ± 2.6	1.1-11.6
Total power (ms^2^)		1303 ± 1734		

**Figure 8:**
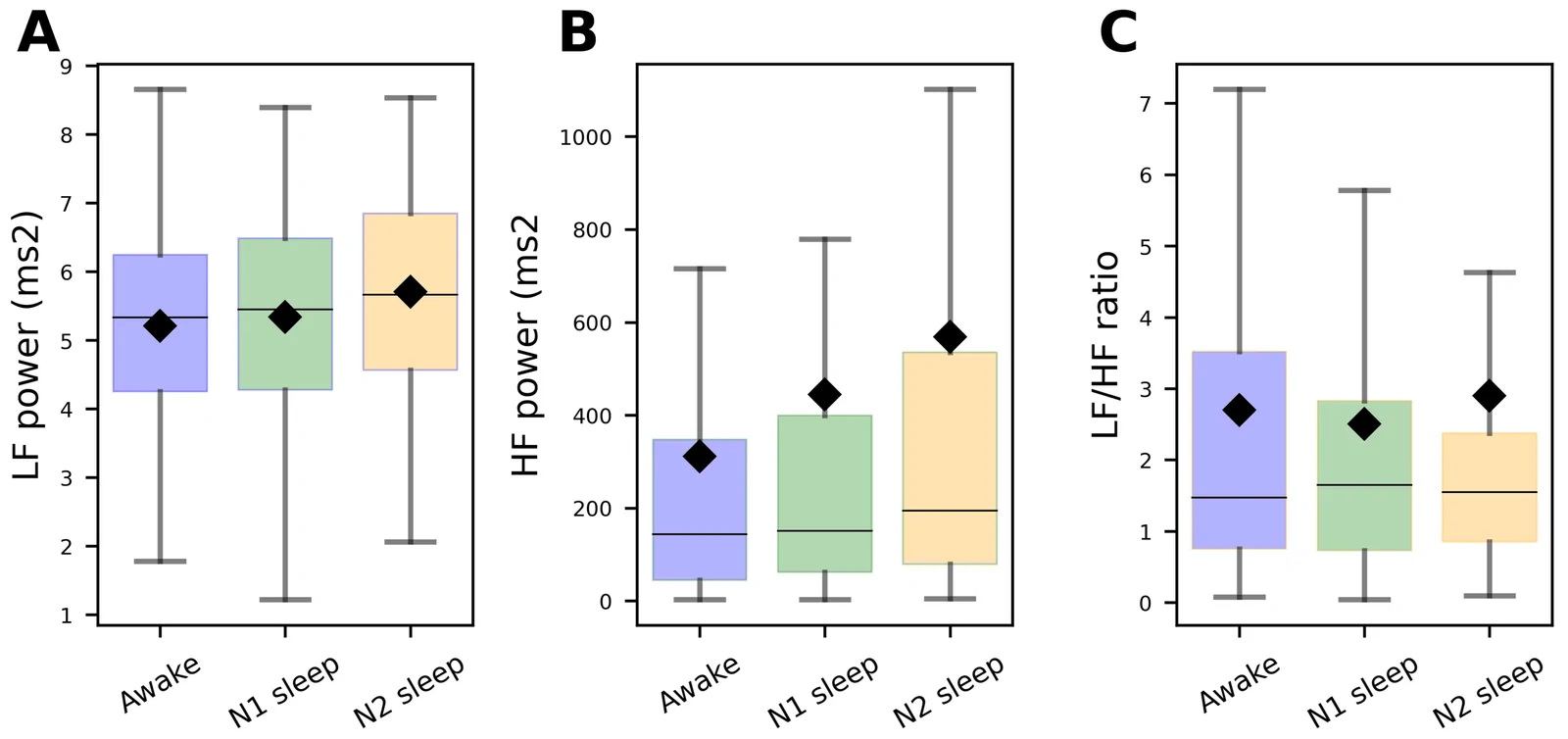
Frequency domain measures of HRV awake and asleep. (A) Mean LF power and (B) mean HF power tend to increase with sleep, but the changes are not statistically significant. There is no significant change in (C) the LF/HF ratio with sleep. N = 95 awake, 76 N1 sleep, and 46 N2 sleep for each graph. The data are highly positively skewed, and outliers are not shown. HRV: heart rate variability; LF: low frequency; HF: high frequency.

We studied the effect of sleep state on frequency domain measures. We found a trend toward sleep increasing HF and LF power during sleep (Figure 8). This trend was not statistically significant. LF, HF, and LF/HF values are highly positively skewed, so for statistical analysis, the data were log-transformed. Single-factor ANOVA of log-transformed LF power versus state had a p-value of 0.23, HF power p = 0.15, and LF/HF ratio p = 0.93.

Inpatient and outpatient EEGs

Of our 103 subjects who had normal sinus rhythm on their ECG, 80 were outpatients and 23 were inpatients. RMSSD of inpatients was 23.6 ± 17.1 (SD), and that of outpatients was higher at 29.1 ± 23.8. However, the variance in both groups is so high that the difference is not statistically significant (Student’s t-test, two-tailed, two-sample unequal variances, p = 0.18). (Illustrating this extreme variation, the patient in Figure 3 with high HRV was an inpatient and the patient with low HRV was an outpatient.) HF of inpatients and outpatients was significantly different. Inpatient HF was 166 ± 204 ms^2^, and that of outpatients was 338 ± 650 (p = 0.04, Student’s t test, two-tailed, unpaired two-sample unequal variances). The lower HF (ms^2^) in inpatients is unlikely to be explained by sex differences, since 13 inpatients were female and 10 were male, and not by age differences, since the average age of inpatients was 50.9 years and that of outpatients was 51.2 years.

## Discussion

In this study, we found that the single-lead ECG recorded during a short-term EEG is fully adequate for recording HRV. All the standard time domain, frequency domain, and nonlinear domain HRV measures could readily be obtained. Compared to reported normal values, we found a reduction in mean HRV. Many individual subjects’ HRV values were extremely low, despite their normal EEG result. We found that both time domain (RMSSD and SDSD) and frequency domain (HF power) measures of HRV were low in this patient population. HRV was reduced in both inpatients and outpatients, but more so in inpatients. Previous studies in healthy normal persons found a significant reduction in HRV with increasing age, and we also found a significant reduction with age in our subjects, but there was no statistically significant effect of biological sex.

HRV normal values

There are several studies that have published normal values for human short-term HRV. The largest of these is by Nunan et al. [17], who did a systematic review of published studies of short-term measures of HRV. The studies they reviewed contained 21,438 participants. Compared to Nunan et al.'s published normal values, our subjects had similar NNI, implying similar heart rates, but their measures of HRV, SDNN, RMSSD, and HF power were reduced.

There are two large studies of short-term HRV in healthy volunteers that show an effect of age and biological sex on HRV. Abhishekh et al. [16] studied short-term HRV in 189 healthy volunteers and reported a marked decline of HRV with aging. Their subjects aged 20 years had a mean RMSSD of approximately 50, but RMSSD declined with increasing age to a value of about 25 by age 60. They also found a strong effect of biological sex, with females having greater HF power. Agelink et al. [15] studied short-term HRV in 309 healthy volunteers and also found an effect of age and sex. RMSSD was 44.7/30.0 (males/females) in the 17-25 age group and declined with every decade of life to 17.8/21.4 (males/females) in their over-55 age group. They did not find a significant sex difference in HF power.

The only HRV measure we could directly compare to both of these previous studies of age effect was RMSSD. Like the healthy normal subjects in those two previous studies, our subjects’ RMSSD and other HRV parameters showed a strong correlation with age; HRV was high in young subjects and much lower in old subjects. Unlike Agelink et al.’s study, in patients undergoing EEGs, there was no statistically significant effect of biological sex, despite a strong trend toward lower HRV in females. The subjects in the two previous studies were specifically selected as healthy normal subjects. Our subjects were clearly not normal because they all had some condition requiring an EEG. Diagnosis and medications were not collected as part of our study, but the patients in our study were having their EEG for a reason, and it is likely that they had a neurological symptom or diagnosis, or medication. The strong effect of age on HRV in all three of these studies suggests that HRV results are difficult to interpret without explicitly considering age.

Other investigators have reported an effect of sleep on HRV. Stein and Pu [10] reviewed many polysomnographic (PSG) studies in which HRV was measured and concluded that HR and HRV progressively decrease during non-REM sleep and increase during REM sleep. In our study, we found instead a relatively small but statistically significant trend toward greater HRV (RMSSD) during non-REM sleep. This was associated with an increase in NNI, implying a decreased pulse rate. The reason for this discrepancy is unclear but may be related to the fact that the sleep we studied occurred during a relatively short EEG compared to the sleep that occurs during overnight PSG, or to the fact that the sleep we observed was during short naps, not the long nighttime sleep that is part of a normal circadian rhythm. We found that HRV measures during any steady state of wakefulness or sleep in a short EEG are relatively stable.

Normal HRV in mammals is related to respiratory sinus arrhythmia (RSA) and to other influences of the parasympathetic and sympathetic nervous systems on the SA node of the heart. In humans, the primary influence is the parasympathetic nervous system through the vagus nerve. Complete blockade of parasympathetic cholinergic activity using atropine markedly increases heart rate, which in the absence of parasympathetic activity is much faster, about 120 beats per minute. Many of our subjects were inpatients at an urban hospital, and all had a health problem or symptom that prompted a clinician to order an EEG. Patients undergoing EEG at our institutions have heterogeneous diagnoses, including encephalopathy, seizures, epilepsy, and other conditions. They are also on a heterogeneous collection of drugs, some of which might influence HRV, such as atropine and other anticholinergics, which can increase heart rate and reduce HRV by blocking parasympathetic action on the heart [19]. We expect that our subjects were similarly heterogeneous with respect to diagnoses and medications and that heterogeneity was reflected in the need to exclude nine of our original 112 patients with normal EEGs because they had an obvious cause for abnormal HRV, like a cardiac pacemaker or atrial fibrillation.

The reasons for reduced HRV in our subjects compared to normal subjects are not clear. The mean of our subjects was -1 SD lower than the mean normal value reported by Nunan et al. [17]. Studies to establish normal HRV values specifically recruited persons with no health conditions and on no medications. None of our subjects would fit those criteria, which could be a reason for reduced HRV. We, like other investigators, found a strong association of increasing age with reduced HRV, so the reduced overall mean HRV in our patients could also be merely because our subjects were older than the subjects in Nunan et al.'s study [17]. Nevertheless, it is clear that having a normal EEG result in no way implies that HRV will also be normal.

We found that HRV of hospital inpatients with normal EEGs was lower than that of outpatients with normal EEGs, and this effect appeared to be independent of sex and age. Lower HRV in inpatients may be a nonspecific indicator of the severity of the acute illness that caused hospitalization, but our data were de-identified, and we could not assess that issue further.

Technical considerations and effect of EEG state

There are some technical factors that could influence HRV. One is the epoch length selected for analysis. Variability in heart rate must be measured over a specific time epoch. HRV studies may be of any length but are commonly classified as “short-term” or “long-term”. Long-term refers to studies with a duration of 24 hours. Short-term usually refers to studies with a duration of five minutes, which is the duration recommended by the 1996 Task Force Report [8]. Five minutes is much longer than a routine 12-lead ECG, but much shorter than the usual EEG. To allow comparison of our results with other published data, we selected five minutes as the epoch duration for HRV analysis. The data were divided into five-minute epochs, with epochs categorized by the EEG “state” of the subject at the time of that epoch. Specific states we analyzed were “awake”, “stage N1 sleep”, and “stage N2 sleep”. Using the American Society for Sleep Medicine guidelines, our results suggest that the state of wakefulness or sleep during a short EEG study has little effect on HRV parameters. However, other investigators doing longer sleep studies have found that sleep does affect HRV, and indeed, have used single-lead ECG by itself to stage sleep [20].

Time domain measures of HRV

Time domain measures describe the statistical variability of the NN intervals during the time epoch. Table 1 shows a list of these measures, along with normal values for short-term recordings of five minutes duration. The most useful and accepted time domain measure is the RMSSD between successive NN intervals in a five-minute epoch. Other measures, such as the number of successive NN intervals with a greater than 50 ms difference, are different, but time domain measures are all correlated with each other. Although we computed all the time domain measures, we did not have a control group and had published normal values only for some of them.

Frequency domain measures of HRV

Time domain measures are useful basic descriptors of the variability of a tachogram, but they ignore its rhythmic structure. The NN intervals are variable, but the variability is not random, it is rhythmic. This problem is addressed by a frequency-domain analysis of the sample. Essentially, the method classifies the rhythmic activity in the tachogram by Fourier analysis of frequency. Standard frequency bands used in short-term HRV studies are a high frequency (HF) band (0.15 to 0.40 Hz) and a low frequency (LF) band (0.04 to 0.15 Hz). The HF band is mainly related to RSA during spontaneous breathing and is a good measure of vagal parasympathetic influences on heart rate. The LF band is thought to be a measure of mixed sympathetic and parasympathetic influences on heart rate. However, during slow-paced breathing at 0.1 Hz (six breaths per minute), the LF band is dominated by RSA [3]. A very low frequency (VLF) band (0.0 to 0.04 Hz) is thought to be dominated by sympathetic and circadian rhythm activity. It is significant in long-term recordings but at present has no known significance in short-term recordings. Nevertheless, VLF band activity may be quite prominent in short-term ECGs (Figure 7).

We found that activity in the HF band was reduced in our subjects. This suggests that vagal parasympathetic activity was impaired. That result could be due to anticholinergic drugs, or to abnormal activity in the medullary nuclei that regulate RSA.

Nonlinear measures of HRV

A final often-used type of HRV analysis is nonlinear measures, which attempt to characterize the degree of nonperiodicity in the sample. A time series measurement may be completely non-random, but the non-random generators of the time series combine to produce a non-periodic, or even “chaotic” result. Nonlinear measures attempt to measure the degree of non-rhythmicity in a tachogram. We did not analyze nonlinear measures during this study.

Potential pitfalls

When measuring HRV, there are a number of potential pitfalls that could possibly occur, which we believe we have minimized in our study. Artifacts are a problem in ECG recording, and they are mainly caused by voluntary muscle and movement artifacts. We minimized the effects of artifacts on ECG by a reliable automated placement of fiducials on each R peak, and then by visual inspection of each placement, with manual adjustment if necessary.

For measurement of autonomic nervous system activity using HRV, ventricular and atrial premature beats constitute artifacts, since they are related to intrinsic activity in the heart conduction pathways downstream from the SA node, and not directly related to autonomic nervous system influences on the SA node. For this reason, RR intervals having atrial and ventricular premature beats were not included in the analysis. Again, we accomplished that by direct visual inspection of QRS complexes. QRS complexes with abnormal morphology are likely to be ventricular beats or aberrantly conducted non-sinus atrial beats, and these were excluded from analysis. Premature atrial beats that clearly lacked a P wave that was normally present were also excluded. The remaining beats having P waves were assumed to originate in the SA node and were included in the analysis. We did not try to assess the morphology of P waves in determining what were or were not atrial premature beats.

“The epileptic heart” and cardiac death in epilepsy

It has long been known that low HRV is predictive of higher morbidity and mortality in patients with known cardiovascular disease [5,9]. In recent years, it has become clear that chronic epilepsy is associated with increased risk of sudden cardiac death [21-23]. Increased risk is apparent at all ages [24]. The reason for this increase is unclear but may be related to ill effects of seizures on the heart, such as catecholamine surges and hypoxemia [6]. Interictal ECG biomarkers for the epileptic heart include such standard measures as Q waves [23] and QT-interval prolongation [25]. With more sophisticated analyses, T wave alternans and increased T wave heterogeneity have been shown to be useful measures [6,26,27], but all these analyses are best performed with prolonged ECG or 12-lead ECG, not with the single-channel ECG performed during EEG.

HRV has been measured in epileptics. Hamdy et al. [28] found an RMSSD of about 20 in two groups of epileptic patients using Holter monitoring. Lotufo et al. [9] did a systematic review and meta-analysis of HRV in epilepsy and antiepileptic drugs. They included 366 studies, and concluded that epileptics had lower HF, SDNN, and RMSSD values compared to controls. HRV abnormalities may have predictive value for SUDEP. Sivathamboo et al. [7] found that HRV was reduced in 31 epileptics who had SUDEP compared to controls. Szurhaj et al. [29] studied the last EEG of 20 SUDEP patients. They found that, unlike non-SUDEP patients, the HR and RMSSD did not change during hyperventilation.

Limitations

We expect our finding that HRV can be reliably measured during routine EEG from the single-channel ECG during the first five minutes of awake state to be robust. The primary limitations are lack of subject diagnoses and lack of age-matched healthy controls, since health status and age both have notable effects on HRV. The main physicians ordering EEGs were neurologists, and we expect epilepsy, cardiac diagnoses, diabetes, and other conditions linked to autonomic nervous system dysfunction to have been present in some or many of our subjects.

## Conclusions

We found that measuring HRV from the single-channel ECG in patients undergoing short-term EEGs is highly feasible. We found that HRV during a short-term EEG is little affected by state of wakefulness, sleep, or time spent in any given state, so a single five-minute HRV study at the beginning of the EEG would be sufficient for a good characterization of HRV. We studied only patients with normal EEGs, so people with common EEG abnormalities such as excessively slow frequencies or epileptiform activity were not included. We expected that persons with normal EEGs, indicating relatively normal functioning of the cerebral cortex, might also have normal HRV, indicating normal function of brainstem cardiovascular and respiratory centers. But that is clearly not the case; even though our subjects had normal EEGs, their average HRV was reduced compared to published normal values, and many subjects had extremely low HRV. Given the growing appreciation of the increased risk of cardiac abnormalities and death in patients with epilepsy, routine analysis of the single-channel ECG done during EEG may be helpful in guiding therapy or better advising patients of their cardiac risk profile. Although HRV analysis can be done at a later time, it would ideally be implemented directly in EEG-reading software so that it can be made part of the routine EEG report.
